# Teaching Neuroimages: Obscured Cerebral Infarction on MRI

**DOI:** 10.1007/s00062-017-0576-x

**Published:** 2017-03-17

**Authors:** Masaaki Hori, Ryusuke Irie, Michimasa Suzuki, Shigeki Aoki

**Affiliations:** 1 Department of Radiology, Juntendo University School of Medicine, 2-1-1 Hongo, Bunkyo-ku, 113-8421 Tokyo, Japan; 2 Department of Radiology, The University of Tokyo, Tokyo, Japan

**Fig. 1:**
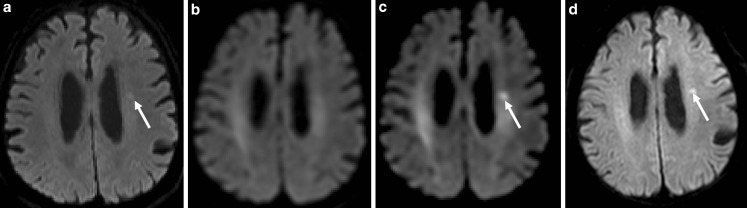
Diffusion-weighted imaging of the brain of the 87-year-old patient showing the acute infarction (*arrow*). **a** DWI (b-value = 1000 s/mm^2^, diffusion time = 22.3 ms), **b** DWI (b-value = 1500 s/mm^2^, diffusion time = 8.5 ms), **c** DWI (b-value = 1500 s/mm^2^, diffusion time = 47.3 ms), **d** DWI (b-value = 1000 s/mm^2^, diffusion time = 22.3 ms) obtained 2 days after the image in Fig. 1a

An 87-year-old man presented with dysarthric speech for 1 day and underwent magnetic resonance imaging (MRI) using a 3-Tesla MRI scanner. Diffusion-weighted imaging (DWI) using a b-value of 1000 s/mm^2^ showed a small abnormality with high intensity on the left side of the deep white matter (Fig. 1a). The DWI using a b-value of 1500 s/mm^2^ and different diffusion times showed that the infarction was obscured in some images (Fig. 1b) and clear in others (Fig. 1c). The effective diffusion time was 8.5 ms for the image shown in Fig. 1b and 47.3 ms for the image shown in Fig. 1c. The infarction was shown clearly in DWI and T2-weighted images obtained during an MRI examination that was completed 2 days after the initial examination (Fig. 1d).

Recent advances in MRI have allowed a reduction in echo time, which has theoretically led to improvement in DWI quality; however, the diffusion time of DWI decreases with the reduction of echo time. Therefore, the utility of this method might be changed, making it inappropriate for the detection of a lesion with restricted water diffusion, such as an acute cerebral infarction. A short diffusion time leads to dramatically reduced diffusion contrast in images of stroke in humans [1]. In the present case, the echo time and diffusion time for the image shown in Fig. 1a are 60 ms and 22.3 ms, respectively. The diffusion time may not be sufficient for the detection of acute cerebral infarction, as in this case.

Previous reports have shown the utility of high b‑value DWI to detect acute ischemic stroke [2, 3]: however, the b‑value itself is not an important factor. As the b‑value used for DWI increases, the diffusion time naturally becomes longer due to hardware limitations of clinical MRI systems. In the presented case, the image shown in Fig. 1b was obtained using a higher b‑value than that shown in Fig. 1a, but the lesion is less clearly demonstrated due to the shorter diffusion time. It should be noted that diffusion time is the key factor for enhancing contrast in DWI.
